# High Performance Polycarbonate Nanocomposites Mechanically Boosted with Titanium Carbide in Material Extrusion Additive Manufacturing

**DOI:** 10.3390/nano12071068

**Published:** 2022-03-24

**Authors:** Nectarios Vidakis, Markos Petousis, Sotirios Grammatikos, Vassilis Papadakis, Apostolos Korlos, Nikolaos Mountakis

**Affiliations:** 1 Mechanical Engineering Department, Hellenic Mediterranean University, Estavromenos, 71410 Heraklion, Greece; vidakis@hmu.gr (N.V.); mountakis@hmu.gr (N.M.); 2 Group of Sustainable Composites, Department of Manufacturing and Civil Engineering, Norwegian University of Science and Technology, 2815 Gjovik, Norway; sotirios.grammatikos@ntnu.no; 3 Institute of Molecular Biology and Biotechnology, Foundation for Research and Technology—Hellas, 71110 Heraklion, Greece; vassilis_papadakis@imbb.forth.gr; 4 Department of Industrial Engineering and Management, International Hellenic University, 14th km Thessaloniki-N. Moudania, Thermi, 57001 Thessaloniki, Greece; apkorlos@ihu.gr

**Keywords:** three-dimensional (3D) printing, additive manufacturing, nanocomposites, polycarbonate (PC), titanium carbide (TiC), fused filament fabrication (FFF), mechanical characterization

## Abstract

Herein, a polycarbonate (PC) polymer is melt extruded together with titanium carbide (TiC) nano powder for the development of advanced nanocomposite materials in material extrusion (MEX) 3D printing. Raw material for the 3D printing process was prepared in filament form with a thermomechanical extrusion process and specimens were built to be tested according to international standards. A thorough mechanical characterization testing course (tensile, flexural, impact, microhardness, and dynamic mechanical analysis-DMA) was conducted on the 3D printed specimens. The effect of the ceramic filler loading was also investigated. The nanocomposites’ thermal and stoichiometric properties were investigated with thermogravimetric analysis (TGA), differential scanning calorimetry (DSC), energy-dispersive X-ray spectroscopy (EDS), and Raman respectively. The specimens’ 3D printing morphology, quality, and fracture mechanism were investigated with atomic force microscopy (AFM) and scanning electron microscopy (SEM) respectively. The results depicted that the addition of the filler decidedly enhances the mechanical response of the virgin polymer, without compromising properties such as its processability or its thermal stability. The highest improvement of 41.9% was reported for the 2 wt.% filler loading, making the nanocomposite suitable for applications requiring a high mechanical response in 3D printing, in which the matrix material cannot meet the design requirements.

## 1. Introduction

Additive manufacturing (AM), in which the 3D printing process belongs, is a technology with high potential for use as a manufacturing process, competing with conventional processes, with which nowadays they can be integrated [1]. Among its advantages, there is the capability to produce complex geometry parts, with several different operating principles, all having a common layer-by-layer manufacturing fashion [1]. Its main weaknesses are related to the materials used and the dimensional accuracy of the produced parts [2]. Hence, a lot of research has been conducted on the investigation of the AM materials’ mechanical performance, i.e., their tensile [3,4,5], compression [6,7,8,9], impact [10,11,12] strength, and their strain rate [13,14]. To induce additional properties to the 3D printing materials, various types of fillers have been used for the development of nanocomposites, making the 3D printing polymers conductive [15,16,17,18], or introducing antimicrobial properties to make them suitable for medical applications [19,20,21,22]. The dimensional accuracy of the AM parts is also investigated in the literature [23].

Polycarbonate (PC) is a thermoplastic polymer used in several engineering applications, such as automotive, data storage, and construction, among others [24]. Due to its characteristics (softness, notch sensitivity of mechanical properties, high melt viscosity), it is not suitable for advanced applications, and to improve its characteristics, it is blended with other thermoplastic polymers, such as PVC [25], or organic and inorganic additives such as nano alumina [26], graphene [27], and multi wall nanotubes [28]. Its blends have been studied and have been made available in the market for many years now [29]. In AM, and especially in the fused filament fabrication (FFF) process, which is employed in this work and is a material extrusion process [30], the applied 3D printing parameters and settings have been studied, towards the optimization of the process [31]. The effect of the 3D printing parameters on the interlayer bonding of the build parts [32,33] and their response under dynamic loading [34], fatigue loading [35], and different strain rates [36] have been presented in the literature. The 3D printed PC parts’ mechanical properties have also been investigated [37], such as impact [38] and creep [39]. The potential for 3D printing parts in orbit with FFF was also investigated [40]. The literature for PC composites and nanocomposites in MEX 3D printing is very limited, with research focusing on nanocomposites with cellulose nanofibers as fillers [41] and carbon fibers [40,42], aiming to enhance the mechanical performance of the matrix material, while the adhesion of the layers and the porosity of the build parts is also investigated [42].

Metal carbides as additives induce advanced properties to the composites, hence they are popular materials used in various types of industrial applications, such as biomedical applications [43], energy storage applications [44], and industrial tools [45]. Titanium carbide (TiC) is a popular metal carbine due to its characteristics, i.e., wear and corrosion resistance, thermal stability, and catalytic characteristics [46]. It has been used as filler to enhance mechanical and electrical properties [47], for many years now [48], in various types of applications such as cutting tools, abrasive-resistant materials [46], energy storage devices [49,50], and optoelectronics [51]. Additionally, mainly in the form of MXenes, it has been used in various types of medical applications for humidity sensors [52] and air purification [53], in films mimicking the human skin [54], and in photothermally killing cancer cells [55]. In AM, the literature is limited, with research focusing mainly on the use of TiC as a filler for the reinforcement of alloys, such as Inconel 718 [56] and Inconel 690 [57,58] in selective laser melting (SLM) [59] for high temperature and corrosion resistance applications.

In this work, for the first time, TiC was used as a filler for the reinforcement of the PC polymer in material extrusion 3D printing. The aim was to produce nanocomposites with enhanced mechanical properties from popular materials in industrial applications. The improved mechanical properties result in decreased parts segments, reduced weight, and material consumption, while, at the same time, exploiting the advantages and characteristics of 3D printing and expanding its use in more advanced industrial areas. Nanocomposites were prepared at various concentrations and tested with tensile, flexural, impact, microhardness, and dynamic mechanical analysis (DMA), according to international standards, to fully characterize their mechanical response. The effect of the filler loading was also investigated. Additionally, the nanocomposites were evaluated for their thermal and spectroscopic behavior and scanning electron microscopy (SEM) was employed for the investigation of the fracture mechanism in the fabricated specimens, while atomic force microscopy (AFM) evaluated the surface quality of the produced filament. In all cases studied, the filler had a positive effect on the mechanical response of the nanocomposites when compared to the pure matrix material, showing the potential of the studied nanocomposites for use in applications requiring an advanced mechanical response. The highest improvement of an enormous 41.9% was recorded in the tensile strength of 2 wt.% filler loading, which had overall the most enhanced performance.

## 2. Materials and Methods

**Figure 1:**
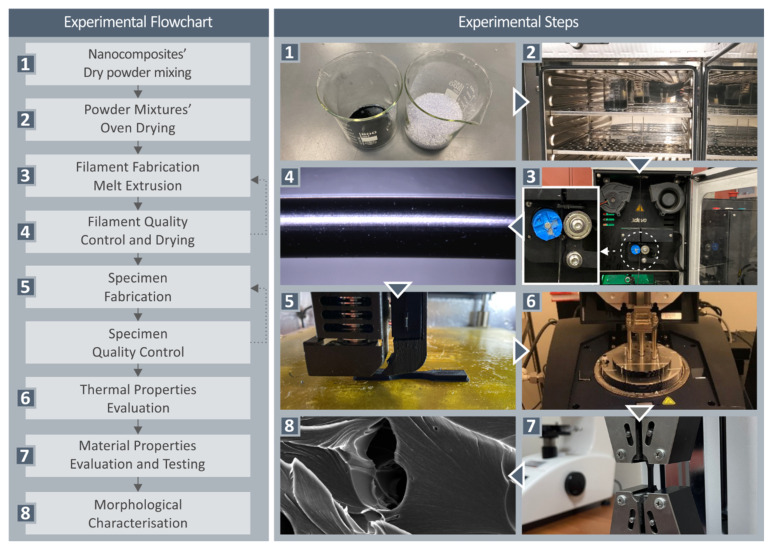
Workflow followed in the current study.

In Figure 1, the workflow of the current study is presented.

### 2.1. Materials

The matrix material of this work was procured in pellet form from Styron Europe GmbH (Horgen, Switzerland). The matrix material was PC polymer under the commercial name EMERGE (PC) 8430-15, produced by Trinseo S.A. (Berwyn, PA, USA). The PC grade, according to its technical datasheet, had a density of 1.20 g/cm^3^, tensile stress at fracture 70.0 MPa, and elongation at fracture 110%. Titanium carbide (TiC) was procured from Nanographi (Ankara, Turkey) in nanopowder/nanoparticles form. The purity of the powder was 99.5+%, the size of the nanoparticles was (NPs) 33–55 nm, they had cubic shape, their true density was 4.5 gr/cm^3^, and their melting point was 3200 °C.

### 2.2. Nanocomposites Fabrication

First, materials (matrix material-PC and filler-TiC) were dried in a laboratory oven (60 °C for 24 h) to remove any humidity from them. Then, the materials were mixed at four different filler loadings, i.e., 1.0 wt.%, 2.0 wt.%, 3.0 wt.%, and 6.0 wt.%. The mixture process was implemented in a glove box to restrain the spread of the powder in the room with a high-power blender. Each mixture was further dried, and then it was fed in a Noztek (Shoreham-by-Sea, UK) single screw extruder. The produced filament was shredded to pellets in a 3devo (Utrecht, The Netherlands) shredder. The pellets were then fed in a 3devo Composer (Utrecht, The Netherlands) single screw extruder for 3D printing 1.75 mm diameter filament production. This extruder was equipped with a screw (rotational speed was set to 4.8 rpm), with a special design for materials and additives mixing, according to the manufacturer, and it had four independent heating zones for better temperature adjustment in the extruder chamber. Temperatures were adjusted to each heating zone based on experiments conducted prior to the production of the filament of this work, and they were as follows: heating zones 1–3 (1 is closer to the nozzle) 200 °C, heat zone 4 240 °C. This process with the two extruders was followed to achieve as good dispersion of the filler in the polymer matrix as possible. Filament, which was the raw material for the 3D printing process that followed, was produced for each nanocomposite studied herein with this process. Filament with the pure PC material was also produced for the manufacturing of 3D printing specimens for comparison purposes.

### 2.3. Specimens Fabrication

**Figure 2:**
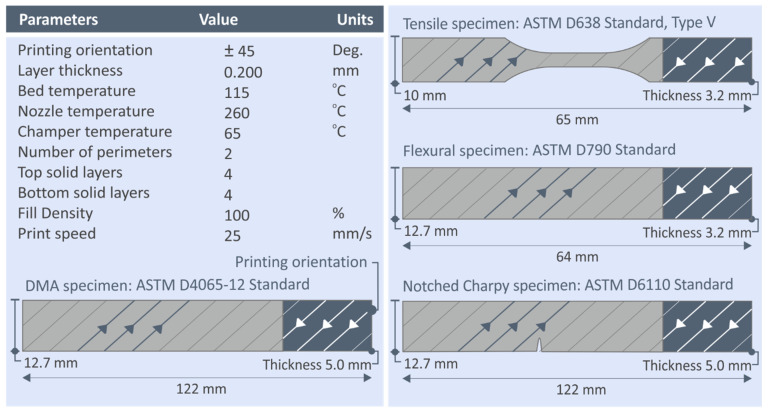
3D printing settings for manufacturing the specimens of this work. Specimens manufactured are shown on the right side of the figure.

As mentioned above, the produced filament was the raw material for the manufacturing of the 3D printed specimens, which was implemented in an Intamsys Funmat HT (Shanghai, China) material extrusion 3D printer. The 3D printing parameters used were the same in all cases, and they were experimentally determined to ensure the processability of the materials before 3D printing the specimens that were tested in this work. The 3D printing parameters used are shown in Figure 2. The Intamsuite software platform (Shanghai, China) was used for the generation of the required G-code files for the 3D printing process. Specimens were manufactured with the 3D printing process according to international standards, as shown in Figure 2. For each nanocomposite material and the pure PC polymer, for each mechanical test, five specimens were manufactured and tested, according to the corresponding standard specifications.

### 2.4. Thermal and Spectroscopic Properties Investigation

Before the mechanical characterization of the prepared nanocomposites, their thermal and spectroscopic properties were investigated to determine characteristic parameters and to evaluate the stability of the nanocomposites. The thermal behavior of the nanocomposites was investigated with thermogravimetric analysis (TGA and differential scanning calorimetry (DSC), while the spectroscopic investigation was conducted with Raman spectroscopy. TGA also verified that the temperatures used for the filament and the specimens’ production during the extrusion processes in this work did not compromise the thermal stability of the materials. The parameters for each test are shown in Table 1.

### 2.5. Nanocomposites Produced Filament Evaluation

The quality of the produced filament was examined before the 3D printing of the specimens with it. During the filament extrusion process, the produced filament diameter was recorded by the 3devo Composer (Utrecht, The Netherlands) extruder, and the produced graphs were inspected to ensure that the diameter was within acceptable deviation for the 3D printing process. Additionally, filament diameter was measured in random positions with a high-quality caliper. The strength of the filament was measured with tensile tests to evaluate its mechanical strength and provide quantitative information regarding the effect of the 3D printing process on the nanocomposites. Tests were conducted on an Imada MX2 (Northbrook, IL, USA) tensile test apparatus, with custom-made grips for the fixture of filament in the machine for the tests. Finally, the surface roughness of the produced filament was measured with atomic force microscopy (AFM) on a MicroscopeSolver P47H Pro (Moscow, Russia), and the resonant frequency was of 300 kHz. These measurements provided an indication of the filament extrusion quality, and the effect of the filler loading on the filament quality could be evaluated.

### 2.6. Mechanical Characterization of the Nanocomposites

For the evaluation of the mechanical performance of the produced nanocomposites in material extrusion 3D printing, as mentioned above, specimens were manufactured to be tested with various mechanical tests according to international standards. The tests conducted are presented in Table 2. In all tests, six specimens were assessed, and all tests were conducted in room temperature conditions.

### 2.7. Morphological Characterization

For the morphological characterization of the specimens, scanning electron microscopy (SEM) was employed. A JEOL JSM 6362LV (Peabody, MA, USA) apparatus was used. Images were taken in high-vacuum mode (20 kV) at different magnification levels on the side surface of tensile specimens to evaluate the 3D printing quality in all filler loadings. The fracture mechanism on the tensile specimens was evaluated by taking images from the corresponding fracture surface of tensile test specimens from all materials prepared and tested in this work. In all cases, specimens were gold coated to avoid charging effects. Energy dispersive X-ray analysis (EDX) was also employed in the same apparatus to verify the basic elements in the nanocomposites. In this case, un-sputtered specimens were used.

## 3. Results

### 3.1. Thermal and Spectroscopic Properties Investigation

**Figure 3:**
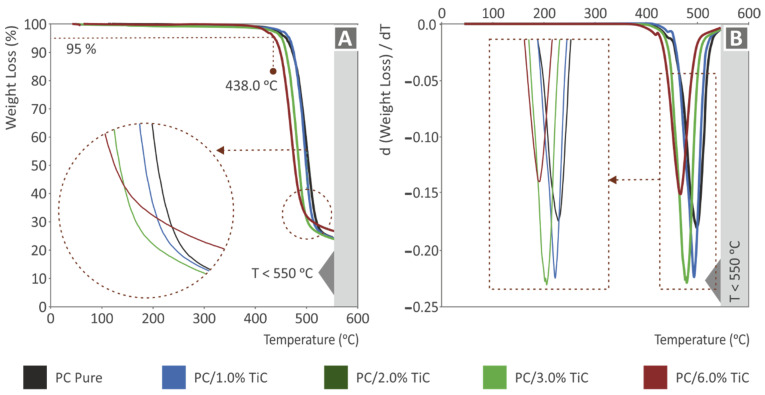
TGA: (**A**) weight (%) vs. temperature (°C) graph for all materials tested; (**B**) weight loss rate (dw/dT) vs. temperature (°C) graph for all materials tested.

Figure 3 shows the thermogravimetric analysis (TGA) results for all materials prepared in this work. Figure 3A shows the weight (%) vs. temperature (°C) graph, which represents the degradation of the materials with the temperature increase. As it is shown, the curves are similar up to the point where the materials start to degrade. This is an indication that the addition of the TiC filler in the PC matrix material did not affect the thermal stability of the polymer at these temperatures. After a critical temperature of about 438 °C, an intense weight loss was observed, and in this region differences between the materials were visible. As the filler increased, the temperature, as the material started to degrade, was reduced, while the complete degradation of the material started at lower temperatures as well. This trend was similar for all materials, and only the highest loading of 6 wt.% slightly differed. Regarding the weight loss rate depicted in Figure 3B, it was increased with the increase of the filler loading, up to 3 wt.%, which exhibited the highest weight loss rate among the materials tested, and it radically decreased for the 6 wt.% loading material, indicating a change in the behavior at this filler concentration and, as expected, a change in the thermal stability of the nanocomposite at higher filler loadings. As the filler concentration increased, the temperature, in which the higher weight loss rate occurred, was reduced.

**Figure 4:**
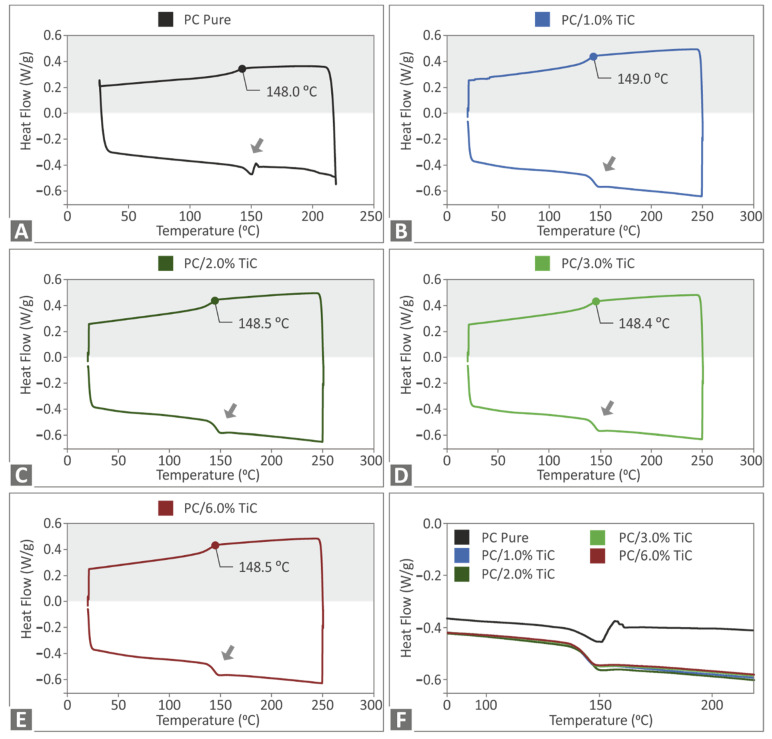
DSC exotherm and endotherm heat flow curve (W/g) to temperature (°C) for: (**A**) pure PC, (**B**) PC/TiC 1 wt.%, (**C**) PC/TiC 2 wt.%, (**D**) PC/TiC 3 wt.%, (**E**) PC/TiC 6 wt.%, and (**F**) comparison of the exotherm curves for all materials tested.

Figure 4 shows the DSC results of the study. In all materials, a similar trend was observed in the materials’ phase change. This indicated a cross-linked structure on the materials. The addition of the filler affected both the endotherm and the exotherm curves of the material. The absorbed energy was reduced when compared with the pure PC material, and rather negligible differences were observed between the different filler loadings in the nanocomposites in the DSC curves.

**Figure 5:**
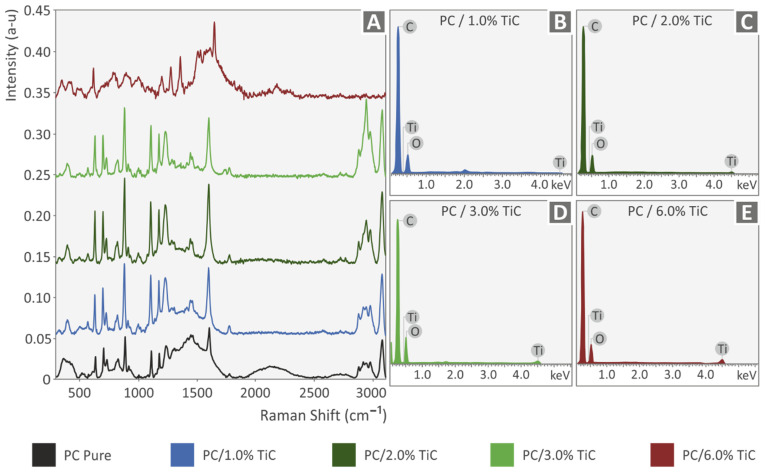
(**A**) Raman spectra for all materials tested, (**B**) EDS graph for PC/TiC 1 wt.%, (**C**) EDS graph for PC/TiC 2 wt.%, (**D**) EDS graph for PC/TiC 3 wt.%, and (**E**) EDS graph for PC/TiC 6 wt.%.

Figure 5A shows the Raman spectra graphs for all materials tested. The highest contribution came from the PC material, while all the nanocomposites exhibited similar curves, except for the 6 wt.%, in which the peaks differed. From the analysis of PC clear, the major Raman peaks were identified, and their related assignments are displayed in the following table x. The range of the Raman peaks found was between 573 cm^−1^ and up to 3073 cm^−1^ (Table 3). Based on the literature, the spectrum measured matched the one of polycarbonate from where the assignments were derived [60,61,62,63].

### 3.2. Nanocomposites Produced Filament Evaluation

**Figure 6:**
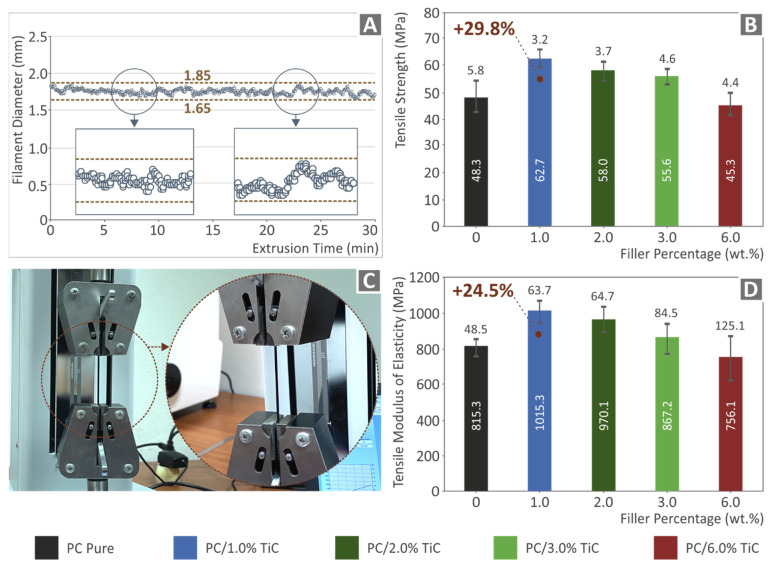
(**A**) Real-time diameter measurement of the produced filament during the filament extrusion process, (**B**) mean tensile strength values and deviation of the produced filament for all materials studied, (**C**) experimental setup for the produced filament tensile tests, and (**D**) mean tensile modulus of elasticity values and deviation of the produced filament for all materials studied.

Figure 6A shows part of the graph of the real-time measurement of the produced filament diameter. As it is shown, the produced filament diameter was kept within a deviation, which is acceptable for the 3D printing process. Figure 6B shows the mean tensile strength values with their deviations on the filament tensile tests. As it is shown, the highest mechanical strength was reported for the 1 wt.% concentration nanocomposite, which was 29.8% higher than the corresponding pure PC material value. At higher filler loadings, the tensile strength decreased, with nanocomposites with filler concentrations up to 3 wt.% having higher strength than the pure PC material and the highest loading of 6 wt.% tested in this work, exhibiting a more inferior response than the pure PC material. Figure 6D shows the corresponding average tensile modulus of elasticity values, which followed the same trend with the tensile strength values, with the 1 wt.% concentration nanocomposite exhibiting 24.5% higher tensile modulus of elasticity than the pure PC material. These results show an initial indication that the addition of this filler significantly enhanced the mechanical response of the matrix material.

**Figure 7:**
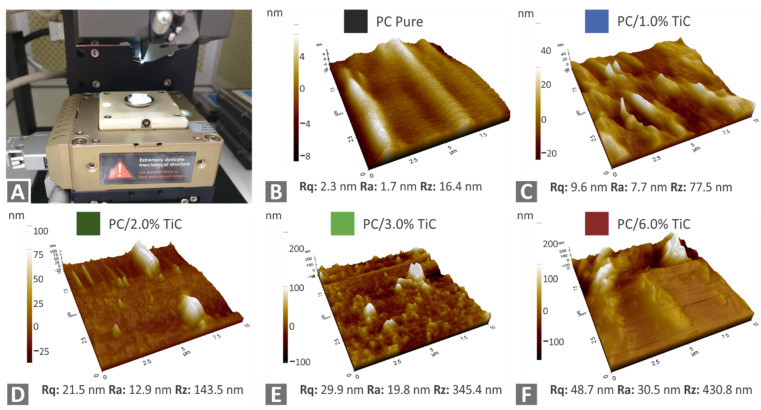
Surface quality measurements with AFM on the produced filament: (**A**) AFM experimental setup, (**B**) pure PC, (**C**) PC/TiC 1 wt.%, (**D**) PC/TiC 2 wt.%, (**E**) PC/TiC 3 wt.%, and (**F**) PC/TiC 6 wt.%.

Figure 7 shows the AFM results from the filament of all materials tested. The topography of the area measured is shown along with the corresponding surface roughness values on the measured surface. As it is shown, the surface roughness increased with the increase of the filler loading.

### 3.3. Mechanical Characterization of the Nanocomposites

**Figure 8:**
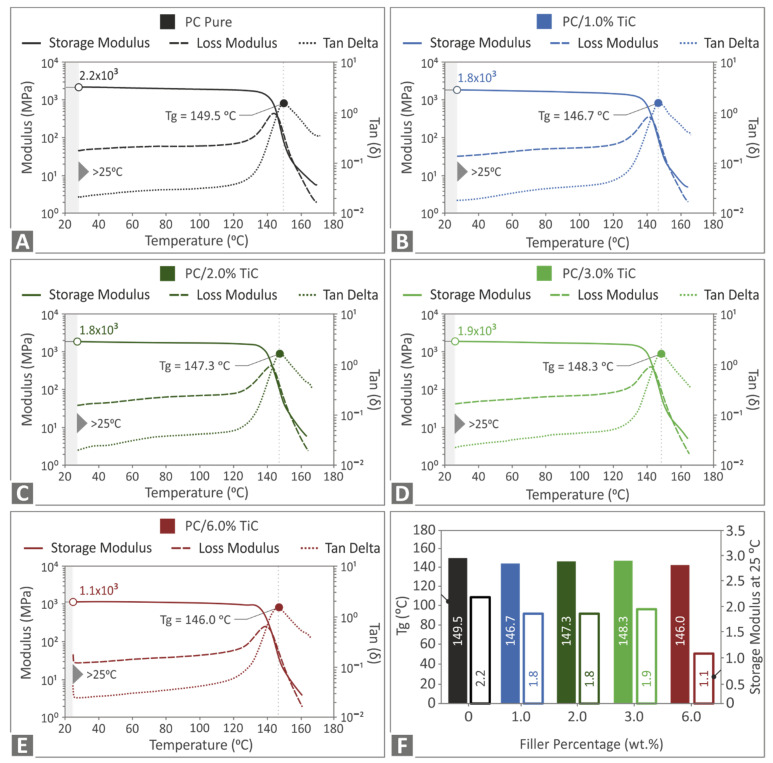
Storage modulus, loss modulus, and tan(delta) graphs for: (**A**) pure PC, (**B**) PC/TiC 1 wt.%, (**C**) PC/TiC 2 wt.%, (**D**) PC/TiC 3 wt.%, (**E**) PC/TiC 6 wt.%, and (**F**) glass transition temperature Tg (°C) and storage modulus values at 25 °C for all materials tested.

Figure 8 presents the storage modulus, loss modulus, and tan(delta) graphs determined in the DMA tests for all materials tested. A similar response was observed for all materials tested for all three values, with the curves having a similar pattern in all corresponding cases. The glass transition temperature was not significantly affected by the addition of the filler in the nanocomposite. Storage modulus values were lower only in the highest concentration of 6 wt.% nanocomposite, showing a less elastic response of the nanocomposite in this case when compared to the other materials of the study. These results indicate that the viscoelastic behavior of the polymer was not affected by the addition of the specific filler.

**Figure 9:**
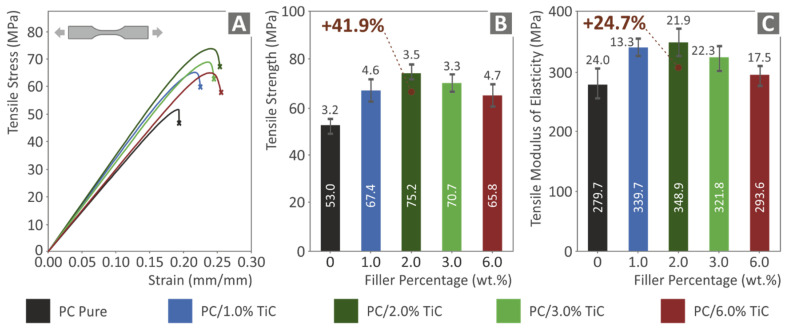
Tensile tests of all materials tested: (**A**) stress vs. strain graphs, (**B**) mean tensile strength and deviation, and (**C**) mean tensile modulus of elasticity and deviation.

Figure 9 presents the tensile test results of the study. Figure 9A shows typical stress vs. strain graphs for all materials tested. It is shown that the addition of the filler in the matrix material increased the ability of the material to deform before failure, making the material stiffer and with higher tensile strength. This is verified in Figure 9B, in which the calculated tensile strength values are presented. In all cases, the nanocomposites exhibited higher tensile strength values when compared with the pure PC polymer. The highest reinforcement was calculated in the nanocomposite with the 2 wt.% filler concentration, which showed an enormous 41.9% increase compared to the corresponding pure PC polymer value. The tensile strength decreased in the nanocomposites with higher filler loadings; still, for the highest loading of 6 wt.% tested in this work, the tensile strength was about 24% higher than the pure PC material. Figure 9C shows the corresponding average tensile modulus of elasticity values, which followed the same trend as the tensile strength values, with the 2 wt.% concentration nanocomposite exhibiting 24.7% higher tensile modulus of elasticity than the pure PC material and the nanocomposite with the highest filler loading of 6 wt.% having 5% higher tensile modulus of elasticity than the pure PC material.

**Figure 10:**
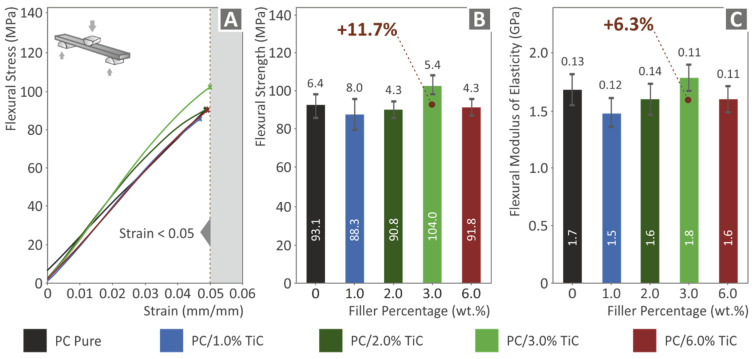
Flexural tests of all materials tested: (**A**) stress vs. strain graphs up to 5% strain following the standard, (**B**) mean flexural strength and deviation at 5% strain, and (**C**) mean flexural modulus of elasticity and deviation at 5% strain.

Figure 10 shows the corresponding flexural test results. The tests were terminated at 5% strain, according to the standard instructions. At low filler loadings, specimens showed inferior responses compared to the pure PC material. The highest calculated value was recorded in the 3 wt.% loading nanocomposite, which exhibited 11.7% higher flexural strength than the pure PC material, showing that this was the optimum loading for the flexural test. At the highest loading of 6 wt.%, the calculated value was lower than the pure PC material. This result, in combination with the remaining results for this filler loading, indicated possible saturation of the filler in this case. Figure 10C shows the corresponding results for the flexural modulus of elasticity. A similar trend with the flexural strength was observed, with the 3 wt.% loading nanocomposite exhibiting a 6.3% increase in the flexural modulus when compared to the pure PC material. The storage modulus presented in Figure 8F can be correlated with the flexural modulus presented in Figure 10C. Comparing these two figures, a similar pattern can be observed with slightly different values, which can be attributed to different specimens, different specimen dimensions, different testing procedures and machines, the anisotropy of the 3D printed specimens, their 3D printing structure, or a random cause.

**Figure 11:**
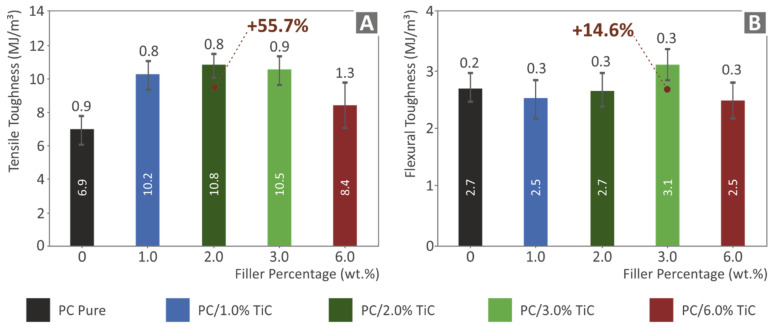
For all materials tested: (**A**) mean tensile toughness (MJ/m^3^) and deviation; (**B**) mean flexural toughness (MJ/m^3^), and deviation.

Figure 11 shows the mean toughness (MJ/m^3^) values and their deviation calculated in the tensile and the flexural tests, respectively. Toughness is a value calculated as an integral of the stress-strain graph and provides an indication of the absorbed energy of the material during the tests. As it is shown, the addition of the filler increased the toughness in the tensile tests in all cases studied, with the highest value calculated in the 2 wt.% loading nanocomposite, which showed a 55.7% increase when compared to the pure PC material. The flexural toughness results followed the trend of the flexural test results, exhibiting the highest flexural toughness value at the 3 wt.% loading nanocomposite, with an increase of 14.6% when compared to the pure PC material.

**Figure 12:**
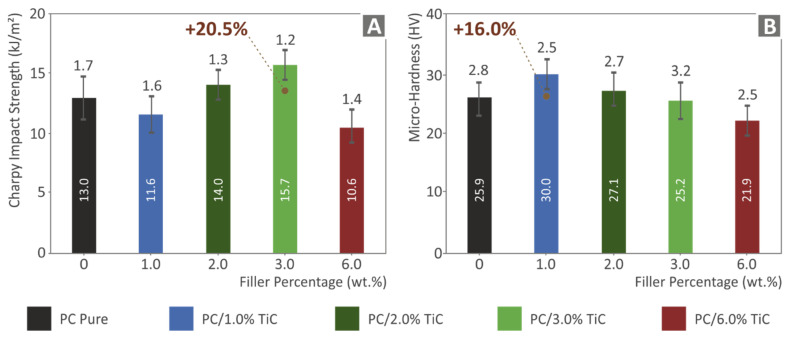
For all materials tested, results for: (**A**) impact strength (kJ/m^2^); (**B**) microhardness.

Figure 12A shows the impact toughness results calculated in the impact tests. A rather similar trend to flexural tests results was observed with inferior impact toughness at lower filler loadings, and the highest impact toughness was calculated for the 3 wt.% nanocomposite, with a 20.5% increase compared to the pure PC material corresponding value. At the highest filler loading, the impact toughness was decreased, with its value lower than the pure PC material. Figure 12B shows the Vickers microhardness measurement results. The introduction of the filler in the polymer matrix increased the microhardness of the material for loadings up to 3 wt.%, and at the highest loading of 6 wt.% studied, the microhardness decreased, with the measured value lower than the corresponding PC material. The highest value was measured in the 1 wt.% nanocomposite, which showed a 16% increase compared to the pure PC material.

### 3.4. Morphological Characterization

**Figure 13:**
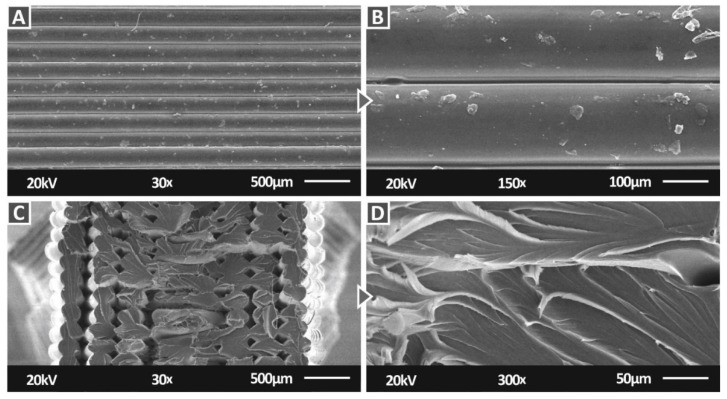
SEM images of the pure PC: (**A**) side surface at 30×, (**B**) side surface at 150×, (**C**) fracture surface at 30×, and (**D**) fracture surface at 300×.

Figure 13 shows SEM images at two magnifications, for the pure PC material on the side and the fracture surface of tensile specimens. On the side surface images (Figure 13A,B), an excellent interlayer fusion can be observed, with no voids or defects visible, showing that the 3D printing settings used were appropriate for the work. In Figure 13B, any abnormalities that are visible on the surface of the filament strands can be attributed to plausible existing material remains in the 3D printer nozzle. In the image of the fracture surface (Figure 13C), a rather brittle failure can be observed, with only a few strands showing deformation. Voids and discontinuities shown on the surface can be attributed to the failure of the specimen during the tensile test. In the higher magnification image (Figure 13D) of the fracture surface, deformation, indicating a ductile behavior on the material, can be observed, so the brittle response of the specimen can be attributed to the 3D printing structure and the effect on the material.

**Figure 14:**
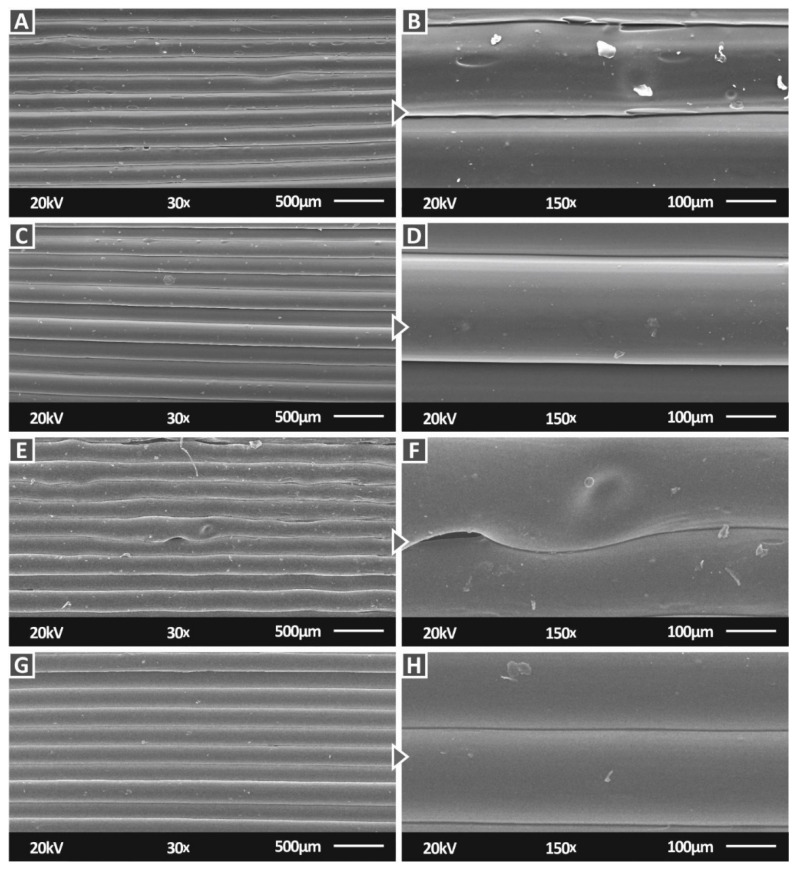
SEM images of the PC/TiC nanocomposites side surface: (**A**) 1 wt.% at 30×, (**B**) 1 wt.% at 150×, (**C**) 2 wt.% at 30×, (**D**) 2 wt.% at 150×, (**E**) 3 wt.% at 30×, (**F**) 3 wt.% at 150×, (**G**) 6 wt.% at 30×, and (**H**) 1 wt.% at 150×.

Figure 14 shows SEM images at two magnifications of the side surface for the PC/TiC nanocomposites for all the filler concentrations prepared in this work. In all cases again, an excellent interlayer fusion can be observed, with no voids or defects visible, verifying here as well that the 3D printing settings used were appropriate for the work. Only in the case of 3 wt.% loading (Figure 14E,F), some imperfections in the 3D printing structure are visible and so was a void between the strands. These can be considered statistically unimportant random issues, attributed to instant changes in the material flow, temperature variation, or filament imperfections, especially since the mechanical performance of this loading nanocomposite was significantly improved compared to the pure PC material.

**Figure 15:**
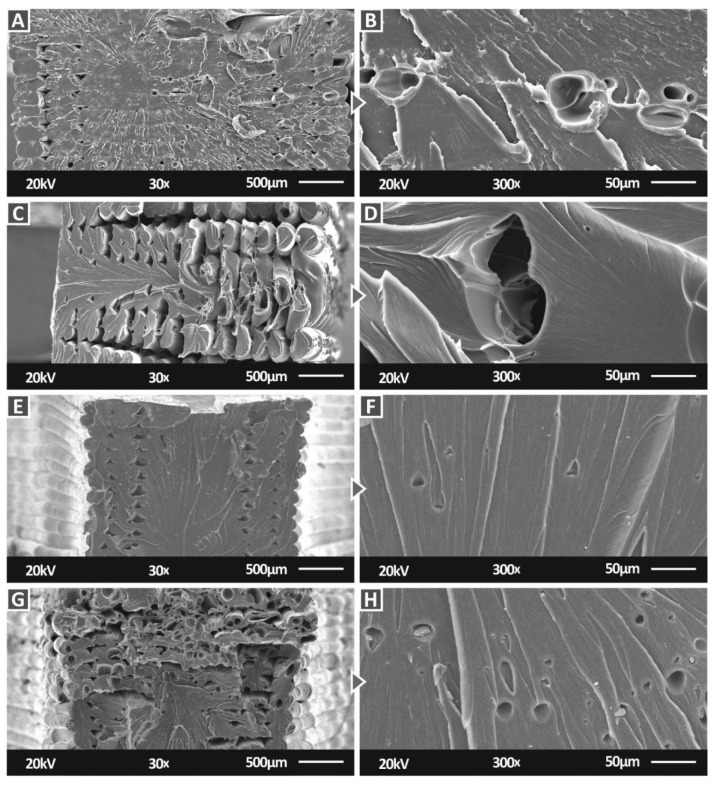
SEM images of the PC/TiC nanocomposites fracture surface: (**A**) 1 wt.% at 30×, (**B**) 1 wt.% at 300×, (**C**) 2 wt.% at 30×, (**D**) 2 wt.% at 300×, (**E**) 3 wt.% at 30×, (**F**) 3 wt.% at 300×, (**G**) 6 wt.% at 30×, and (**H**) 1 wt.% at 300×.

Figure 15 shows SEM images at two magnifications of the fracture surface for the PC/TiC nanocomposites for all the filler concentrations prepared in this work. The 1 wt.% and 3 wt.% loading nanocomposites (Figure 15A,E) exhibited a brittle fracture mechanism with no visible deformation in the fracture surface. At the 2 wt.% and 6 wt.% loading (Figure 15C,G), the 3D printing structure collapsed during the break of the specimen on the tensile test. At the 2 wt.% loading, any visible voids could be attributed to the failure of the specimen. Part of the fracture surface showed no deformation, indicating a brittle behavior, while in the remaining fracture surface, a more ductile response was observed with visible deformation on the filament strands. In the 6 wt.% loading, voids were visible in the filament strands, possibly due to air gaps in the material. These deficiencies contributed to the reduced mechanical response of the specific loading; the same for the 2 wt.% loading, part of the fracture surface showed no deformation, indicating a brittle behavior, while in the remaining fracture surface, a more ductile response was observed. The strands with air gaps were in this area. The higher magnification images (Figure 15B,D,F,H) agreed with the corresponding lower magnification images (Figure 15A,C,E,G) regarding the brittleness or the ductility of the material. At the 1 wt.% loading, the material has a brittle behavior, although it was not so brittle as the 3 wt.% case. In the other two filler loadings (2 wt.% and 6 wt.%) a more ductile material response was observed, with voids again visible in the 6 wt.% loading.

**Figure 16:**
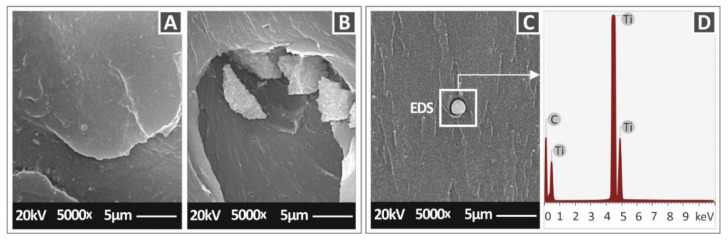
SEM images of the PC/TiC nanocomposites fracture surface at 5000× magnification: (**A**) 1 wt.%, (**B**) 6 wt.%, (**C**) 3 wt.%, and (**D**) EDS graph of the 3 wt.%, image taken on the agglomeration shown in (**C**).

Figure 16A–C, presents higher magnification images of 5000× for the 1 wt.%, 6 wt.%, and 3 wt.%, respectively. No agglomerations were visible in the 1 wt.% loading. In the 3 wt.% loading, minimum agglomerations were observed, while in the 6 wt.% case, agglomerations were detected in the SEM images, verifying a possible saturation threshold for this filler loading and an insufficient dispersion of the filler in the matrix material, which had a reported negative impact on the mechanical response of the specific nanocomposite. In Figure 16D, the EDS graph taken in the agglomeration area of the 3 wt.% loading is shown. As expected, the presence of titanium (Ti) in the material was verified, with a high peak indicating a high concentration of this element in the examined region of the specimen.

## 4. Discussion

**Figure 17:**
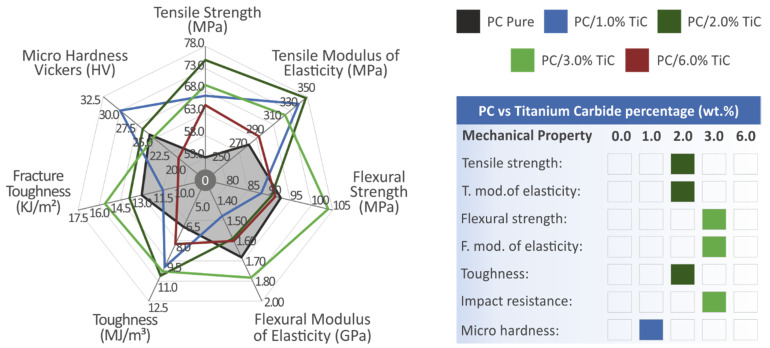
Summary of the mechanical tests’ results. The shaded area in the spider graph depicts the pure PC material’s mechanical properties. On the right side, the filler loading that exhibited the highest performance on each test is shown.

PC/TiC nanocomposites were prepared in this work for MEX 3D printing, and various tests were conducted for the mechanical characterization of the prepared materials. For loadings up to 3 wt.%, the addition of the filler had a positive impact on the mechanical response of the material compared to the pure PC polymer, with the 2 wt.% exhibiting the highest values in the tensile tests and an enormous 41.9% improvement compared to the matrix material, and the 3 wt.% had the highest values in the flexural tests, with 11.7% compared to the matrix material. In the highest filler loading studied of 6 wt.%, the mechanical performance of the material decreased, showing that this loading is plausibly approaching (but not reaching) a saturation threshold for the TiC filler in the PC polymer. The 6 wt.% is not the threshold, since at the tensile tests, the calculated value was still higher than the matrix material, but in most of the tests, the response of this specific loading was degraded compared to the rest of the materials studied. Figure 17 summarizes the mechanical results of the tests conducted in this work.

In the tensile tests of the 3D printed specimens, the pure PC exhibited lower tensile strength (53 MPa) than the nominal of the material, reported in its datasheet (60 MPa). Such differences are expected and can be attributed to the 3D printing process and differences in the experimental conditions. With the addition of the filler, the tensile strength of the nanocomposites reached the nominal tensile strength of the pure PC material and was higher than the pure material in the case of 2 wt.%, showing that the nanocomposites prepared in the work can exploit the advantages of 3D printing in applications requiring advanced mechanical performance. In literature, there is no similar study with PC material and TiC in AM or any other type of manufacturing process since this filler is mainly used as a reinforcement in metals, so the results of the study cannot be evaluated with literature. In metals, the reported reinforcement is similar to the results of this study [47].

For the mechanical characterization of the nanocomposites developed in this work, specimens were 3D printed with a MEX process, so the effect of 3D printing in the nanocomposites was also considered, along with the effect of the filler loading. For the 3D printing of the specimens, the filament was fabricated with a thermomechanical extrusion process from raw materials (polymer and TiC nanoparticles) in powder form. The filament was also tested in the tensile experiment to qualitatively evaluate the effect of the 3D printing process on the material. The results followed the same trend as the tests on the 3D printed specimens, with an enhancement in the tensile strength in all loadings tested when compared to the matrix material. Still, results cannot be directly compared to the tensile tests on the 3D printed specimens, since the tensile tests on the filament were not following a standard.

In all cases studied, the thermal stability of the matrix material was not affected by the addition of the filler, while the temperatures used for the filament extrusion and the manufacturing of the specimens were not close to the degradation temperatures of the matrix material and the nanocomposites. EDS confirmed the elements in the nanocomposites, while the SEM images revealed a very good 3D printing quality and a rather brittle fracture mechanism in most cases in the tensile tests. One critical parameter in the preparation of nanocomposites is the dispersion of the filler in the matrix material, to form a proper NPs network. In this work, a two-step process was followed to achieve as good a dispersion as possible, while agglomerations were observed mainly in the 6 wt.% loading nanocomposite in the SEM images. Additionally, in all tests conducted, the deviation between the results was within acceptable limits, with the filler significantly reinforcing the PC polymer, which is an indication of a well-distributed NPs network, as a result of a good dispersion of the filler in the matrix.

The comparison of the samples with the PC-clear showed that Raman peaks have the following changes. An increase in the PC-TiC samples is presented in the phenyl ring vibration (631 cm^−1^), the C-H out-of-plane bending (700 cm^−1^), ν[O-(C=O)-O], ν(C-CH3), r(CH3), ν(ring) (883 cm^−1^), and the C-O-C stretching (1106, 1175, 1231 cm^−1^). There was a gradual increase versus concentration in the CH2vAS or C-H (2912 cm^−1^) and the C-H stretching (3071 cm^−1^). Furthermore, two new Raman peaks were identified in the Phenyl ring vibration (647 cm^−1^) and the C=O vibration (1740 cm^−1^). Lastly, the peaks of CH2vAS/C-H and CH/CH2 stretch modes remained constant. The described peaks are also shown in the following Table 4.

## 5. Conclusions

In this work, and for the first time, PC/TiC nanocomposites at various filler loadings were prepared for MEX 3D printing to investigate the effect of the filler as a reinforcement in the polymer matrix in materials exploiting the advantages of the AM technology. The thermal and spectroscopic properties of the nanocomposites were investigated, while their morphological characteristics were studied with AFM and SEM. The results indicated that TiC as a filler in NPs form can significantly reinforce the PC material for loadings up to 3 wt.%. Due to the porosity that 3D printing introduces to the parts, their mechanical properties are decreased compared to the bulk material properties. The addition of the filler in the matrix material can overcome this effect in the mechanical properties of the 3D printed parts. These nanocomposites are not alternatives to the corresponding injection molded parts, but each material and process is suitable for specific types of applications. It was also found that higher loadings had a negative impact on the mechanical response of the nanocomposites. PC is a medium- to high-performance polymer widely used in many industries and is becoming more and more popular nowadays in 3D printing. The addition of the nanomaterial enhanced the mechanical performance, creating a potential for further expanding 3D printing applications with the use of such materials.

With the thermomechanical extrusion process followed, the processability of the materials was flawless, making the process easily adapted in a higher-scale industrial environment. So, the nanocomposites prepared in this work are suitable for applications requiring an enhanced mechanical response in MEX 3D printing, in which the strength of the materials is one of the main weaknesses of the process. Additionally, this is an affordable process since the matrix material for laboratory-scale costs 0.04 EUR/gr and the filler 1.18 EUR/gr, while considering that it is used in low concentrations, the additional cost in the raw materials for the process is not important. For the 3 wt.%, the materials cost raises from 0.04 EUR/gr for the pure material to about 0.075 EUR/gr, which is an important increase in the materials cost (of about 87%), but the effect in the overall cost of the process is low. Additionally, such costs can be considerably decreased for industrial-scale use. As future work, different carbides can be used as fillers in the PC polymer, and additional properties of the prepared nanocomposites can be investigated based on the properties of the filler, which are induced in the matrix in the nanocomposites.

## Figures and Tables

**Table 1:** Parameters for the thermal and spectroscopic investigation.

TGA	
Instrument	Perkin Elmer Diamond TGA/DTGA (Waltham, MA, USA)
Temperature range	40 °C to 550 °C
Temperature ramp	10 °C/min
Atmosphere	Nitrogen
DSC	
Instrument	TA Instruments DSC 25 (New Castle, DE, USA)
Temperature sweep	25–225–25 °C (5 min at 225 °C)
Temperature rate	15 °C/min
Raman	
Instrument	LabRAM HR Raman spectrometer (HORIBA Scientific, Kyoto, Japan)
Laser	Solid-state
Laser power	90 mW
Power on the sample	40 mW
Center wavelength	532 nm
Lens	50×, 10.6 long working distance, 0.5 numerical aperture (LMPlanFL N, Olympus, Tokyo, Japan)
Laser spot diameter	1.7 μm
Axial focal length	2 μm
Spectra resolution	2 cm^−1^
Acquisition duration	10 s
Acquisition range	300–3100 cm^−1^

**Table 2:** Tests that were conducted in the work for the mechanical characterization of the prepared nanocomposites.

DMA	
Instrument	TA Instruments rheometer (DHR 20) (TA Instruments, New Castle, DE, USA)
Standard	ASTM D4065-12
Test	Three-point-bending
Preload	0.1 N
Oscillation amplitude	30 μm
Frequency	1 Hz
Temperature range	30–200 °C
Temperature rate	5 °C/min
Tensile	
Instrument	Imada MX2 (Northbrook, IL, USA)
Standard	ASTM D638-02a
Specimen	Type V with 3.2 mm thickness
Testing speed	10 mm/min
Flexural	
Instrument	Imada MX2 (Northbrook, IL, USA)
Standard	ASTM D790
Support span	52 mm
Testing speed	10 mm/min
Impact	
Instrument	Terco MT 220 (Kungens Kurva, Sweden)
Standard	ASTM D6110
Release height	367 mm
Specimens	Notched
Microhardness	
Instrument	Innova Test 300 (Maastricht, The Netherlands)
Method	Vickers
Indentations’ duration	10 s
Applied load	200 gF

**Table 3:** Major Raman peaks identified and their related assignments.

Wavenumber (cm^−1^)	Raman Peak Assignment
573	Phenyl ring vibration
633	Phenyl ring vibration
703	C-H out-of-plane bending
731	C-H out-of-plane bending
826	Phenyl ring vibration
886	ν[O-(C=O)-O], ν(C-CH3), r(CH3), ν(ring)
1109	C-O-C stretching
1176	C-O-C stretching
1234	C-O-C group asymmetric vibration
1602	Phenyl ring vibration
2874	CH2vS or C-H
2912	CH2vAS or C-H
2971	CH/CH2 stretch modes polarized
3073	C-H stretching

**Table 4:** The Raman spectra behavioral differences between the clear PC and the TiC samples.

Wavenumber (cm^−1^)	Assignment	Change
631	Phenyl ring vibration	Increased vibration for TiC samples
647	Phenyl ring vibration	New peak for TiC samples
700	C-H out-of-plane bending	Increase of peak for TiC samples
883	ν[O-(C=O)-O], ν(C-CH3), r(CH3), ν(ring)	Increase of peak for TiC samples
1106	C-O-C stretching	Increase of peak for TiC samples
1175	C-O-C stretching	Increase of peak for TiC samples
1231	C-O-C group asymmetric vibration	Increase of peak for TiC samples
1740	C=O vibration	New peak for TiC samples
2912	CH2vAS or C-H	Gradual increase vs. TiC concentration
2911	CH2vAS or C-H	Constant
2970	CH/CH2 stretch modes polarized	Constant
3071	C-H stretching	Gradual increase vs. TiC concentration

## Data Availability

The data presented in this study are available upon request from the corresponding author.
